# Effect of TongXie-YaoFang on Cl^−^ and HCO_3_^−^ Transport in Diarrhea-Predominant Irritable Bowel Syndrome Rats

**DOI:** 10.1155/2016/7954982

**Published:** 2016-06-14

**Authors:** Xiaofang Lu, Shengsheng Zhang, Cheng Yang, Zhengfang Wang, Luqing Zhao, Zhenyu Wu, Jing Xie

**Affiliations:** Digestive Disease Center, Beijing Hospital of Traditional Chinese Medicine Affiliated to Capital Medical University, Beijing 100010, China

## Abstract

TongXie-YaoFang (TXYF) can effectively alleviate the symptoms of diarrhea-predominant irritable bowel syndrome (D-IBS) patients. However, the curative mechanism has not been fully clarified. The study was designed to investigate the effect of TXYF on the colonic ion transport induced by serotonin (5-HT) in D-IBS rats. A method of multiple stress (neonatal maternal separation (NMS) combined with restraint stress (RS)) was used to induce the D-IBS model. The model rats were randomly divided into two groups: NMS + RS group and TXYF-formula group, and the normal control (no handling) rats were classified as NH group. In the NMS + RS group, the change of short-circuit current (Δ*I*_sc_) induced by 5-HT was lower than that in the NH and TXYF-formula groups. After removing of the extracellular Cl^−^ or HCO_3_^−^ or basolateral Na^+^ or blocking the cystic fibrosis transmembrane conductance regulator (CFTR), Na^+^-K^+^-2Cl^−^ cotransporter (NKCC), Na^+^-HCO_3_^−^ cotransporter, Cl^−^/HCO_3_^−^ exchanger, K^+^ channel, or Na^+^/K^+^-ATPase, respectively, there was no difference in 5-HT-induced Δ*I*_sc_ among the three groups. These data suggest that TXYF can regulate 5-HT-induced Cl^−^ and HCO_3_^−^ secretion, possibly mediated by the combined action of CFTR, NKCC, Na^+^-HCO_3_^−^ cotransporter, Cl^−^/HCO_3_^−^ exchanger, K^+^ channel, and Na^+^/K^+^-ATPase.

## 1. Introduction

Diarrhea-predominant irritable bowel syndrome (D-IBS) is a chronic functional gastrointestinal disease, which is characterized by abdominal pain/discomfort and diarrhea without the demonstrable pathological evidence according to Rome III criteria [1]. As one of the most frequent subtypes of IBS patients, the D-IBS patients have lower quality of life than the other subtypes [2, 3]. However, as a multifactorial disease, the pathogenesis of D-IBS is complex and has not been fully understood as yet. Hence, the therapeutic effect has not been satisfactory for patients, although multiple medications have been developed as the optional therapies including loperamide, bile acid sequestrants, antispasmodics, tricyclic antidepressants, and rifaximin [4–9]. Currently, a series of randomized controlled trials have shown that Traditional Chinese Medicine (TCM) such as the representative formula TongXie-YaoFang (TXYF) can significantly alleviate the symptoms and improve the quality of life in D-IBS patients [10, 11]. However, the mechanism of the therapeutic effect has not been known, which limits the application of TXYF on a larger scale.

Serotonin (5-HT) is an important monoamine neurotransmitter in the brain-gut axis, which makes a great contribution to the onset and development of D-IBS. The hyperactivity of 5-HT has been found in D-IBS patients, which is recognized to be related with the disturbed visceral sensitivity, epithelium secretion, and smooth muscle contraction [12–14]. In the physiological studies, it has been shown that 5-HT as a potent secretagogue produces a significant effect on the intestinal ion transport including Cl^−^ or HCO_3_^−^ secretion via multiple ion channels, cotransporters, and exchangers such as the cystic fibrosis transmembrane conductance regulator (CFTR), Na^+^-K^+^-2Cl^−^ cotransporter (NKCC), Na^+^-HCO_3_^−^ cotransporter, Cl^−^/HCO_3_^−^ exchanger, or K^+^ channel, all of which are related to the creation of osmotic pressure for dragging fluid into the enteric cavity [15, 16]. At present, much work has confirmed the changes of 5-HT-induced intestinal ion transport under stress [17] or in diarrheal diseases [18]. However, in D-IBS as a disease which is closely related to chronic stress and could be manifested by the symptom of diarrhea [19], it still remains unclear whether the altered ion transport induced by 5-HT is present. Further whether TXYF-formula can work on the ion transport also needs to be explored. Hence, the aims of this study were to investigate the effect of TXYF on 5-HT-induced ion transport in D-IBS rats and meanwhile explore the related mechanism.

## 2. Materials and Methods

### 2.1. Animals

Sprague-Dawley male rats (postnatal day 1) were obtained from Vital River Laboratories Animal Technology Co., Ltd. (Beijing, China), and kept in Dongzhimen Hospital Affiliated to Beijing University of Chinese Medicine. All the rats were housed under a 12-hour light/dark cycle (lights on at 8:00 a.m.) with a standard temperature (21–23°C) and humidity (50%  ± 5%) and had access to food and water ad libitum. In this study, female rats were excluded to eliminate the possible effects of estrogen or the other female hormones on the secretory and sensory responses in the intestine [20]. All manipulations were performed between 8:00 a.m. and 11:00 a.m. every day to minimize the influence of circadian rhythms. On postnatal day 22, all the litters were weaned and kept in the individual cages with 3-4 pups per cage.

The study was performed according to the institutional ethical guidelines and conformed to the requirements of the Institutional Animal Care and Use Committee of Beijing University of Chinese Medicine. All animal care and experimental procedures were approved by the Animal Ethics Committee of Dongzhimen Hospital Affiliated to Beijing University of Chinese Medicine.

### 2.2. Neonatal Maternal Separation (NMS) Combined with Restraint Stress (RS)

On postnatal days 2–21, the neonatal maternal separation was conducted as previously described [21, 22]: the NMS + RS litters were removed from their home cages and separated from their maternal rats for 3 hours each day. During the period of separation, the litters stayed alone in the compartments, where the temperature was maintained at 23 ± 0.5°C in a thermally regulated facility. The litters were returned to their home cages immediately after separation. The normal control litters remained in their home cages with their maternal rats all the time.

On postnatal days 50–59, the restraint stress was performed as follows [23, 24]: the NMS + RS rats were placed in a transparent plastic restraint cylinder (4 × 4 × 18 cm^3^) for 3 hours, in which they could move forward and backward but could not turn around.

### 2.3. TXYF Composition and Administration

TXYF-formula was composed of the following traditional Chinese herbal medicines as recorded by the “Formulas of Chinese Medicine” [25]: Baizhu (Rhizoma Atractylodis Macrocephalae) 93.75 g, Baishao (Radix Paeoniae Alba) 62.5 g, Chenpi (Pericarpium Citri Reticulatae) 46.875 g, and Fangfeng (Radix Saposhnikoviae) 31.25 g, which were composed in 6 : 4 : 3 : 2 proportion. All raw materials in the formula were examined according to the quality control criteria in the* Chinese Pharmacopoeia* [26]. It was manufactured by the Preparation Room for TCM of the Beijing Hospital of Traditional Chinese Medicine.

The NMS + RS rats were divided into two groups (NMS + RS group and TXYF-formula group), and the normal control (no handling) rats were classified as NH group. From postnatal day 60, the rats in the TXYF-formula group were given intragastric administration with TXYF-formula (4.92 g/100 g/d). And the NH group and NMS + RS group were given distilled water. The delivery volumes in each group were always 2 mL/100 g/d, serving twice a day, for 14 consecutive days.

### 2.4. Reagents and Apparatuses

The preparation of Krebs-Henseleit solution (K-HS, in mmol/L) was as follows: NaCl 117, KCl 4.7, MgCl_2_ 1.2, NaHCO_3_ 24.8, KH_2_PO_4_ 1.2, CaCl_2_ 2.56, and glucose 11.1; K-HS without Cl^−^: NaCl, KCl, MgCl_2_, and CaCl_2_ were replaced by sodium gluconate, potassium gluconate, magnesium gluconate, and calcium gluconate, respectively; K-HS without HCO_3_^−^: NaHCO_3_ was replaced by NaCl and the solution was buffered with 10 mmol/L HEPES-free acid, bubbled with 100% O_2_; K-HS without Cl^−^ and HCO_3_^−^: sodium gluconate took the place of NaCl and NaHCO_3_ and potassium gluconate, magnesium gluconate, and calcium gluconate took the place of KCl, MgCl_2_, and CaCl_2_, respectively, with being buffered with 10 mmol/L HEPES and gassed with 100% O_2_; K-HS without Na^+^: N-methyl-D-glucamine chloride (NMDGCl) and choline HCO_3_ took the place of NaCl and NaHCO_3_, respectively. If needed, the osmolality was adjusted to 290 mOsmol/L with D-mannitol.

The following were used: 5-HT, batch number: 1001156278; indomethacin, batch number: 1001087688; N-phenylanthranilic acid (DPC), batch number: 101078880; CFTR(inh)-172, batch number: 6311; glibenclamide, batch number: 1001068037; 4,4′-diisothiocyano-2,2′-stilbenedisulfonic acid (DIDS), batch number: 1001339605; bumetanide, batch number: 101016760; amiloride, batch number: 101093389; 4-acetamido-4′-isothiocyanatostilbene-2,2′-disulfonic acid (SITS), batch number: 1001208418; BaCl_2_, batch number: 1398; ouabain, batch number: 101354363. All reagents were purchased from Sigma-AldrichCo.

A multichannel voltage-current clamp (VCC MC6) was purchased from Physiologic Instruments Corporation; the bridge amplifier (ML228) and the recording and analysis system for the physiological data (Power Lab) were purchased from AD Instruments Corporation.

### 2.5. Tissue Preparation

After treatment, all the rats in the three groups were killed with the method of cervical dislocation. The distal colon (6-7 cm from the anus) was removed quickly, cut longitudinally along the mesenteric border, and flushed with ice-cold K-HS. The tissue with the mucosal side down was fixed onto a petri dish with silica gel in the bottom. The petri dish was filled with ice-cold K-HS containing 95% oxygen and 5% carbon dioxide. The serosal, muscular, and submucous layers were carefully separated from the mucosal layer with fine tweezers under a microscope. Then the mucosal layer was cut into small sheets with areas of more than 0.5 cm^2^ for further measurement. Two sheets could be obtained from one segment of the distal colon.

### 2.6. Ussing Chamber Experiment

The mucosal preparations from the distal colon were mounted in the Ussing chamber to measure the short-circuit current (*I*_sc_). K-HS (5 mL) was injected into the two adjacent compartments of Ussing chamber, respectively, with circulating 95% oxygen and 5% carbon dioxide; the pH was maintained at 7.35–7.45 and the temperature was at 37°C. The tissues were left to incubate for 60 min to stabilize the electrical parameters. The voltage across the tissues was clamped to zero and the basal *I*_sc_ was measured. After adding the drugs or blockers to the basolateral or apical side for 30 min, 5-HT (10 *μ*mol L^−1^) was added to the basolateral side. *I*_sc_ were recorded continuously by a chart recorder. The changes in *I*_sc_ (Δ*I*_sc_) were calculated to evaluate the ion transport by the difference value before and after 5-HT stimulation and were normalized as current per unit area of mucosa (*μ*A/cm^2^). To measure the transmembrane resistance, electrical stimulation of 1 mV was applied and the resistance was calculated according to Ohm's Law. Indomethacin (10 *μ*mol L^−1^) was routinely added to the basolateral side of the tissue to block the influence of endogenous prostaglandins in the experiment [27].

### 2.7. Statistical Analysis

The statistical analyses were performed by using SPSS 17.0 software (SPSS, Chicago, IL, USA) and each value was expressed as means ± SEM. All the original data in the study was distributed normally and conformed to homogeneity of variance. The differences among the three groups were analyzed using one-way analysis of variance (ANOVA) followed by least-significant difference (LSD) test to compare the differences between two groups. *P* < 0.05 was considered statistically significant.

## 3. Results

### 3.1. Effect of TXYF on the Basic Electrophysiological Properties and 5-HT-Induced ΔI_sc_

**Figure 1:**
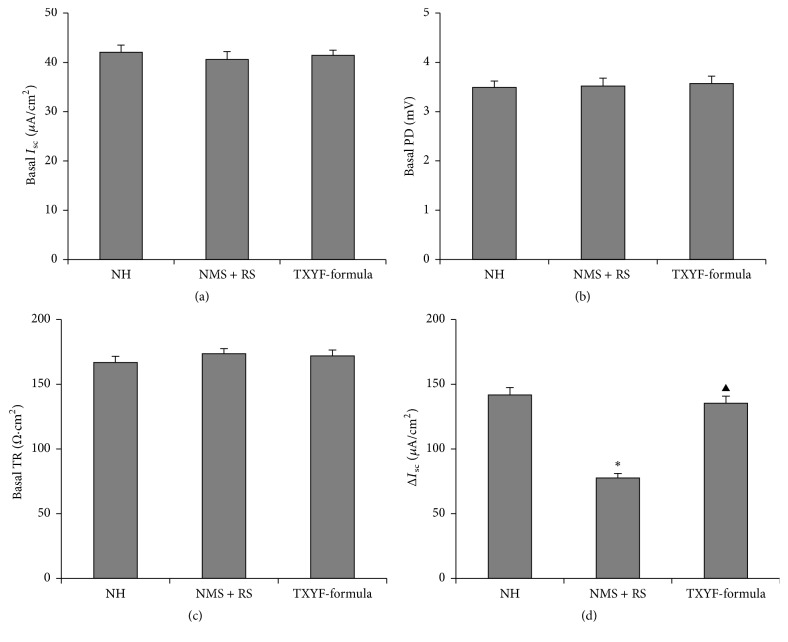
Comparison of basal electrophysiological properties and 5-HT-induced Δ*I*_sc_ of colonic mucosa among the three groups (means ± SEM, *n* = 18). Basal *I*_sc_ (a); basal potential difference (PD) (b); basal transmembrane resistance (TR) (c); Δ*I*_sc_ induced by 5-HT (d). ^**∗**^ *P* < 0.01 versus NH group; ^▲^ *P* < 0.01 versus NMS + RS group.

There were no significant differences in the basal *I*_sc_, potential difference (PD), and transmembrane resistance (TR) among the three groups (*P* > 0.05, Figures 1(a)–1(c)). After addition of 5-HT (10 *μ*mol L^−1^) in the basolateral side, Δ*I*_sc_ in the NMS + RS group was reduced more than that in the NH group; after treatment with TXYF, Δ*I*_sc_ in the TXYF-formula group was significantly increased compared with the NMS + RS group (*P* < 0.01, Figure 1(d)).

### 3.2. Effects of TXYF on 5-HT-Induced ΔI_sc_ after Removal of Cl^−^, HCO_3_^−^, or Na^+^ from K-HS

**Figure 2:**
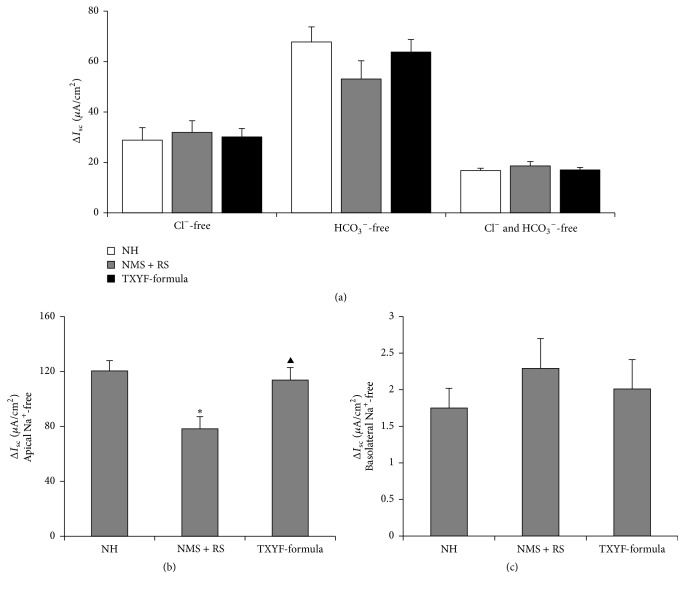
Comparison of 5-HT-induced Δ*I*_sc_ of colonic mucosa in Cl^−^-free, HCO_3_^−^-free, or Cl^−^- and HCO_3_^−^-free (a); apical Na^+^-free (b); basolateral Na^+^-free (c) K-HS among the three groups (means ± SEM, *n* = 6). ^*∗*^ *P* < 0.01 versus NH group; ^▲^ *P* < 0.05 versus NMS + RS group.

When the Cl^−^ and HCO_3_^−^ were removed from K-HS, respectively, there was no significant difference in 5-HT-induced Δ*I*_sc_ among the three groups; in the Cl^−^- and HCO_3_^−^-free K-HS, no significant difference was found among the three groups as well (*P* > 0.05, Figure 2(a)). In addition, after removing the apical Na^+^ from K-HS, 5-HT-induced Δ*I*_sc_ was lower in the NMS + RS group compared with the NH group (*P* < 0.01, Figure 2(b)) and TXYF-formula group (*P* < 0.05, Figure 2(b)). In contrast, when the Na^+^-free K-HS was applied to the basolateral side of the colonic mucosa, no significant difference was found in 5-HT-induced Δ*I*_sc_ among the three groups (*P* > 0.05, Figure 2(c)).

### 3.3. Effects of TXYF on the 5-HT-Induced ΔI_sc_ after Application of the Channel Blockers

**Figure 3:**
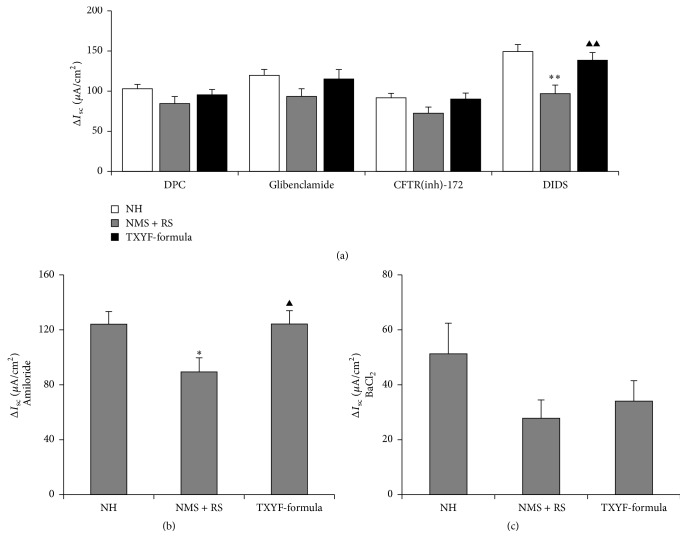
Comparison of 5-HT-induced Δ*I*_sc_ of colonic mucosa after apical application of DPC, a nonselective Cl^−^ channel blocker; glibenclamide or CFTR(inh)-172, the cAMP-independent Cl^−^ channel blockers; and DIDS, a Ca^2+^-independent Cl^−^ channel blocker (a); apical application of amiloride, an epithelial Na^+^ channel blocker (b); basolateral application of BaCl_2_, a K^+^ channel inhibitor (c) among the three groups (means ± SEM, *n* = 6). ^*∗*^ *P* < 0.05 and ^*∗∗*^ *P* < 0.01 versus NH group; ^▲^ *P* < 0.05 and ^▲▲^ *P* < 0.01 versus NMS + RS group.

Following the apical application of a nonselective Cl^−^ channel blocker, namely, DPC (1 mmol L^−1^), or glibenclamide (1 mmol L^−1^) or CFTR(inh)-172 (100 *μ*mol L^−1^), known as the cystic fibrosis transmembrane conductance regulator (CFTR) blockers, Δ*I*_sc_ induced by 5-HT had no significant differences among the three groups (*P* > 0.05, Figure 3(a)). In contrast, after a Ca^2+^-dependent Cl^−^ channel (CaCC) blocker, namely, DIDS (500 *μ*mol L^−1^), was added to the apical side, 5-HT-induced Δ*I*_sc_ remained lower in the NMS + RS group than that in the TXYF-formula group and NH group (*P* < 0.01, Figure 3(a)). Similarly, with the apical application of amiloride (100 *μ*mol L^−1^), an epithelial Na^+^ channel (ENaC) blocker, Δ*I*_sc_ induced by 5-HT was higher in NH and TXYF-formula groups than that in NMS + RS group (*P* < 0.05, Figure 3(b)). However, after basolateral application of the K^+^ channel inhibitor, namely, BaCl_2_ (5 mmol L^−1^), there was no statistical difference in 5-HT-induced Δ*I*_sc_ among the three groups (*P* > 0.05, Figure 3(c)).

### 3.4. Effects of TXYF on the 5-HT-Induced ΔI_sc_ after Application of the Cotransporter or Exchanger Inhibitors

**Figure 4:**
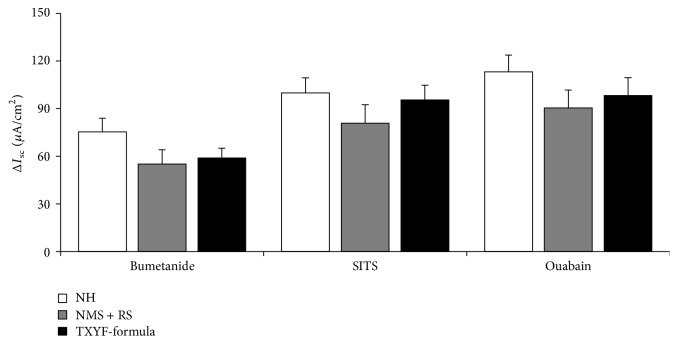
Comparison of 5-HT-induced Δ*I*_sc_ of colonic mucosa in the basolateral presence of Na^+^-K^+^-2Cl^−^ cotransporter (NKCC) inhibitor, bumetanide; Na^+^-HCO_3_^−^ cotransporter or Cl^−^/HCO_3_^−^ exchanger inhibitor, SITS; Na^+^/K^+^-ATPase inhibitor, ouabain, among the three groups (means ± SEM, *n* = 6).

In the presence of bumetanide (100 *μ*mol L^−1^) in the basolateral side, an inhibitor of Na^+^-K^+^-2Cl^−^ cotransporter (NKCC), there was no significant difference among the three groups in Δ*I*_sc_ induced by 5-HT (*P* > 0.05, Figure 4). After the basolateral addition of SITS (100 *μ*mol L^−1^), an inhibitor of Na^+^-HCO_3_^−^ cotransporter or Cl^−^/HCO_3_^−^ exchanger, Δ*I*_sc_ induced by 5-HT had no significant difference among the three groups (*P* > 0.05, Figure 4). Similarly, after the basolateral side was treated with ouabain (1 mmol L^−1^), a Na^+^/K^+^-ATPase inhibitor, Δ*I*_sc_ was statistically insignificant among the three groups (*P* > 0.05, Figure 4).

## 4. Discussion

In the present study, it was found that TXYF could regulate the secretory maladjustment of Cl^−^ and HCO_3_^−^ in D-IBS rats; when the colonic mucosa was pretreated with the inhibitors of CFTR, NKCC, Na^+^-HCO_3_^−^ cotransporter, Cl^−^/HCO_3_^−^ exchanger, K^+^ channel, or Na^+^/K^+^-ATPase, no difference between TXYF and NMS + RS groups was found, indicating that TXYF could regulate the anion transport via these elements as CFTR, NKCC, Na^+^-HCO_3_^−^ cotransporter, Cl^−^/HCO_3_^−^ exchanger, K^+^ channel, and Na^+^/K^+^-ATPase.

TXYF as a popular formula in TCM was first recorded in * Dan Brook Heart Law*, which is a comprehensive medical book edited in Yuan Dynasty. Four Chinese herbs are included in the prescription, which are Baizhu (three Taels), Baishao (two Taels), Chenpi (one and a half Taels), and Fangfeng (one Tael). TXYF is a representative formula for antidiarrhea and analgesia and recognized to be an alternative therapy for D-IBS. A clinical study performed by Pan et al. [10] showed that TXYF could significantly relieve the abdominal pain/discomfort and improved the stool property in D-IBS patients. A recent animal experiment also demonstrated that TXYF could increase the pain threshold and decrease the number of fecal pellets in IBS rats [28]. However, the curative mechanism has not been clear although much work has been done.

At present, although the pathogenesis of D-IBS has not been understood clearly, the dysfunction of the brain-gut axis has been recognized as one of the most important pathophysiologies, which is mainly regulated by a variety of brain-gut peptides including 5-HT [29]. In D-IBS patients, increased platelet-depleted plasma 5-HT concentration under fasting and fed conditions was found [13]. Besides, in an animal experiment, the 5-HT level in the distal colon, spinal cord, and hypothalamus rose in D-IBS model rats as well [30], which demonstrated that the upregulated level of 5-HT contributed to the onset of D-IBS. In addition to regulating intestinal motility and sensitivity, 5-HT as a potent intestinal secretagogue plays an important role in maintaining the balance of intestinal electrolyte and fluid, which is related to the occurrence of diarrhea [31]. In our study, when exogenous 5-HT was added to the basolateral side of colonic mucosa, *I*_sc_ increased in all the three groups. Besides, in NMS + RS group, a decline in Δ*I*_sc_ was elicited compared with the NH group. The result could be supported by another in vitro study conducted by Goldhill et al. [32], in which it was found that the wrap restraint stress rats displayed a reduced response to 5-HT. Further, after treatment with the TXYF-formula, the mucosal response to 5-HT was restored to the similar level to that observed in NH group, which showed that TXYF-formula was able to regulate the alteration of colonic secretion induced by 5-HT in D-IBS rats.

However, interestingly, the secretion induced by 5-HT in the colonic mucosa of the NMS + RS group was lower than that in the NH group and TXYF-formula group, which seemed contradictory to the hypersecretion of diarrhea. Consider the possible reason that may be attributed to the differences between in vivo and in vitro tests and related to increase of 5-HT level or desensitization of 5-HT receptors in the D-IBS rats. As in our previous study, the increased 5-HT level and decreased 5-HT_4_ receptor expression in D-IBS rats have been demonstrated [33], but the specific mechanism needed further discussion.

It is well known that, under normal conditions, Cl^−^ transport is the key link in maintaining the balance of the intestinal electrolyte, which is secreted into the enteric cavity and generates the osmotic driving force for water [34]. The general process of Cl^−^ secretion has been elaborated by *Frizzell and Hanrahan* [35]: the Cl^−^ enters across the basolateral membrane mediated by NKCC and is secreted into the enteric cavity from the apical side via cAMP- and Ca^2+^-dependent Cl^−^ channels. In the present study, we found that TXYF-formula was able to regulate the secretion of Cl^−^ induced by 5-HT. The supporting evidence was displayed as follows: (1) in the normal K-HS, 5-HT-induced Δ*I*_sc_ in the NMS + RS group differed significantly from that in the TXYF-formula group, but when the extracellular Cl^−^ was removed, the difference disappeared; (2) after the apical application of CFTR blockers or basolateral application of NKCC inhibitor in the tissue, Δ*I*_sc_ had no statistical difference between the NMS + RS group and TXYF-formula group. Besides, these results also showed that CFTR and NKCC participated in the effect of TXYF on the colonic Cl^−^ transport. However, when the CaCC was blocked, the statistical difference between the NMS + RS group and TXYF-formula group still remained, which implied that TXYF-formula regulated 5-HT-induced Cl^−^ secretion via cAMP-dependent Cl^−^ channels instead of CaCC. The inference was supported by a physiological experiment, in which it was shown that CaCC had no effect on the 5-HT-induced Cl^−^ secretion in the rat colon [15].

In a recent experiment, it has been shown that HCO_3_^−^ secretion induced by 5-HT was present in the rat colon [16]. In our study, in addition to the regulation of Cl^−^ secretion, we found that TXYF also altered HCO_3_^−^ secretion in the colonic mucosa. When the extracellular HCO_3_^−^ was removed or Na^+^-HCO_3_^−^ cotransporter or Cl^−^/HCO_3_^−^ exchanger was blocked, 5-HT-induced Δ*I*_sc_ did not differ between the NMS + RS group and TXYF-formula group. Besides, the same result was obtained when the extracellular Cl^−^ and HCO_3_^−^ were both removed. Thus, it was demonstrated that TXYF-formula could alter the colonic secretion by regulating the transport of both Cl^−^ and HCO_3_^−^.

Besides, we further explored more possible regulatory mechanisms of TXYF on the anion secretion and found that, in addition to the specific channels, transporters, and exchangers, the transport of K^+^ and basolateral Na^+^ were involved in the secretion of Cl^−^ and HCO_3_^−^, which was demonstrated by the following evidence. (1) The previous study has reported that the basolateral K^+^ conductance can maintain the electrical driving force for the anion secretion [35]. In our study, in the presence of the nonselective K^+^ channel blocker (BaCl_2_), the 5-HT-induced Δ*I*_sc_ was similar between the NMS + RS group and TXYF-formula group, which suggested that the effect of TXYF on anion secretion might be related with the basolateral K^+^ transport. However, because at least two types of K^+^ channels had been found, the precise regulatory subtypes needed further studies. (2) In addition to the K^+^ transport, it was also found that the basolateral Na^+^ participated in the process, which was associated with the generation of electrochemical driving force for anion secretion [36]. As shown in our study, after removal of the basolateral Na^+^ or in the presence of the Na^+^-K^+^-ATPase inhibitor (ouabain), 5-HT-induced Δ*I*_sc_ did not differ between the TXYF-formula and NMS + RS groups, indicating that the regulation of TXYF on anion secretion was partly dependent on the basolateral Na^+^ transport.

Moreover, in our study, we also investigated the effect of TXYF on Na^+^ absorption. After removal of the apical Na^+^ or application of the epithelial Na^+^ channel blocker (amiloride), Δ*I*_sc_ still differed between the NMS + RS group and TXYF-formula group. Therefore, it can be seen that TXYF-formula had little effect on the Na^+^ absorption. That was probably because, in the physiological condition, the Na^+^ absorption played a tiny role in the 5-HT-induced secretory response in the rat colon, as reported by Ning et al. [15].

In conclusion, the present study has demonstrated that TXYF was able to regulate 5-HT-induced Cl^−^ and HCO_3_^−^ secretion in D-IBS-like rats, possibly mediated by the transport elements as CFTR, NKCC, Na^+^-HCO_3_^−^ cotransporter, Cl^−^/HCO_3_^−^ exchanger, K^+^ channel, and Na^+^/K^+^-ATPase, at least in part.

## References

[1] Drossman D. A. (2007). Introduction. The Rome Foundation and Rome III. *Neurogastroenterology and Motility*.

[2] Yao X., Yang Y. S., Cui L.-H. (2012). Subtypes of irritable bowel syndrome on Rome III criteria: a multicenter study. *Journal of Gastroenterology and Hepatology*.

[3] Singh P., Staller K., Barshop K. (2015). Patients with irritable bowel syndrome-diarrhea have lower disease-specific quality of life than irritable bowel syndrome-constipation. *World Journal of Gastroenterology*.

[4] Nee J., Zakari M., Lembo A. J. (2015). Current and emerging drug options in the treatment of diarrhea predominant irritable bowel syndrome. *Expert Opinion on Pharmacotherapy*.

[5] Lävo B., Stenstam M., Nielsen A.-L. (1987). Loperamide in treatment of irritable bowel syndrome—a double-blind placebo controlled study. *Scandinavian Journal of Gastroenterology*.

[6] Odunsi-Shiyanbade S. T., Camilleri M., McKinzie S. (2010). Effects of chenodeoxycholate and a bile acid sequestrant, colesevelam, on intestinal transit and bowel function. *Clinical Gastroenterology and Hepatology*.

[7] Martínez-Vázquez M. A., Vázquez-Elizondo G., González-González J. A., Gutiérrez-Udave R., Maldonado-Garza H. J., Bosques-Padilla F. J. (2012). Effect of antispasmodic agents, alone or in combination, in the treatment of Irritable Bowel Syndrome: systematic review and meta-analysis. *Revista de Gastroenterologia de Mexico*.

[8] Xie C., Tang Y., Wang Y. (2015). Efficacy and safety of antidepressants for the treatment of irritable bowel syndrome: a meta-analysis. *PLoS ONE*.

[9] Kane J. S., Ford A. C. (2016). Rifaximin for the treatment of diarrhea-predominant irritable bowel syndrome. *Expert Review of Gastroenterology & Hepatology*.

[10] Pan F., Zhang T., Zhang Y.-H., Xu J.-J., Chen F.-M. (2009). Effect of Tongxie Yaofang Granule in treating diarrhea-predominate irritable bowel syndrome. *Chinese Journal of Integrative Medicine*.

[11] Huang P.-R., Dai F.-H. (2008). Clinical observation on Tong Xie Yao Fang pulsing for the treatment of 56 cases of diarrhea predominant irritable bowel syndrome. *Journal of Traditional Chinese Medieine*.

[12] Houghton L. A., Atkinson W., Whitaker R. P., Whorwell P. J., Rimmer M. J. (2003). Increased platelet depleted plasma 5-hydroxytryptamine concentration following meal ingestion in symptomatic female subjects with diarrhoea predominant irritable bowel syndrome. *Gut*.

[13] Atkinson W., Lockhart S., Whorwell P. J., Keevil B., Houghton L. A. (2006). Altered 5-hydroxytryptamine signaling in patients with constipation- and diarrhea-predominant irritable bowel syndrome. *Gastroenterology*.

[14] Moskwa A., Boznańska P. (2007). Role of serotonin in the pathophysiology of the irritable bowel syndrome. *Wiadomości Lekarskie*.

[15] Ning Y., Zhu J.-X., Chan H.-C. (2004). Regulation of ion transport by 5-hydroxytryptamine in rat colon. *Clinical and Experimental Pharmacology and Physiology*.

[16] Kaji I., Akiba Y., Said H., Narimatsu K., Kaunitz J. D. (2015). Luminal 5-HT stimulates colonic bicarbonate secretion in rats. *British Journal of Pharmacology*.

[17] Li Y., Li L. S., Zhang X. L., Zhang Y., Xu J. D., Zhu J. X. (2015). An enhanced cAMP pathway is responsible for the colonic hyper-secretory response to 5-HT in acute stress rats. *Physiological Research*.

[18] Turvill J. L., Connor P., Farthing M. J. G. (2000). The inhibition of cholera toxin-induced 5-HT release by the 5-HT_3_ receptor antagonist, granisetron, in the rat. *British Journal of Pharmacology*.

[19] Zhu L., Huang D., Shi L. (2015). Intestinal symptoms and psychological factors jointly affect quality of life of patients with irritable bowel syndrome with diarrhea. *Health and Quality of Life Outcomes*.

[20] Söderholm J. D., Yates D. A., Gareau M. G., Yang P.-C., MacQueen G., Perdue M. H. (2002). Neonatal maternal separation predisposes adult rats to colonic barrier dysfunction in response to mild stress. *American Journal of Physiology—Gastrointestinal and Liver Physiology*.

[21] Ren T.-H., Wu J., Yew D. (2007). Effects of neonatal maternal separation on neurochemical and sensory response to colonic distension in a rat model of irritable bowel syndrome. *American Journal of Physiology—Gastrointestinal and Liver Physiology*.

[22] Lee J., Kim J. Y., Jahng J. W. (2014). Highly palatable food during adolescence improves anxiety-like behaviors and hypothalamic-pituitary-adrenal axis dysfunction in rats that experienced neonatal maternal separation. *Endocrinology and Metabolism*.

[23] Xu J. R., Luo J. Y., Shang L., Kong W. M. (2006). Effect of change in an inhibitory neurotransmitter of the myenteric plexus on the pathogenetic mechanism of irritable bowel syndrome subgroups in rat models. *Chinese Journal of Digestive Diseases*.

[24] van Zyl P. J., Dimatelis J. J., Russell V. A. (2016). Behavioural and biochemical changes in maternally separated Sprague-Dawley rats exposed to restraint stress. *Metabolic Brain Disease*.

[25] Deng Z. J. (2003). *Formulas of Chinese Medicine*.

[26] Pharmacopoeia Commission of the Ministry of Public Health of PRC (2005). *Chinese Pharmacopoeia*.

[27] Yang N., Tian Y.-M., Zhang X.-H. (2008). A dual role of 5-hydroxytryptamine receptor 3 in serotonin induced ion transport in rat distal colon. *European Journal of Pharmacology*.

[28] Shi H.-L., Liu C.-H., Ding L.-L. (2015). Alterations in serotonin, transient receptor potential channels and protease-activated receptors in rats with irritable bowel syndrome attenuated by Shugan decoction. *World Journal of Gastroenterology*.

[29] Kennedy P. J., Cryan J. F., Dinan T. G., Clarke G. (2014). Irritable bowel syndrome: a microbiome-gut-brain axis disorder?. *World Journal of Gastroenterology*.

[30] Sun J.-H., Wu X.-L., Meng Y.-F. (2015). Electro-acupuncture decreases 5-HT, CGRP and increases NPY in the brain-gut axis in two rat models of Diarrhea-predominant irritable bowel syndrome(D-IBS). *BMC Complementary and Alternative Medicine*.

[31] Cirillo C., Vanden Berghe P., Tack J. (2011). Role of serotonin in gastrointestinal physiology and pathology. *Minerva Endocrinologica*.

[32] Goldhill J., Porquet M.-F., Angel I. (1998). The response of rat colonic mucosa to 5-hydroxytryptamine in health and following restraint stress. *European Journal of Pharmacology*.

[33] Wang Z.-F., Guo Q.-K., Zhang S.-S. (2013). Effects of Shuganjianpi prescription on the 5-HT and its receptors of colon mucosa in D-IBS rats. *Beijing Journal of Traditional Chinese Medicine*.

[34] Hodges K., Gill R. (2010). Infectious diarrhea: cellular and molecular mechanisms. *Gut Microbes*.

[35] Frizzell R. A., Hanrahan J. W. (2012). Physiology of epithelial chloride and fluid secretion. *Cold Spring Harbor Perspectives in Medicine*.

[36] Skou J. C. (2004). The identification of the sodium pump. *Bioscience Reports*.

